# Cost-effectiveness of pretreatment HIV drug resistance testing in people living with HIV in Iran

**DOI:** 10.1371/journal.pone.0309528

**Published:** 2024-09-06

**Authors:** Hossein Mirzaei, Abedin Iranpour, Firooz Esmaeilzadeh, Mohsen Barouni, Fatemeh Mehrabi, Ebrahim Ranjbar, Hamid Sharifi

**Affiliations:** 1 HIV/STI Surveillance Research Center, and WHO Collaborating Center for HIV Surveillance, Institute for Futures Studies in Health, Kerman University of Medical Sciences, Kerman, Iran; 2 Department of Public Health, School of Public Health, Maragheh University of Medical Sciences, Maragheh, Iran; 3 Health Service Management Research Center, Institute for Futures Studies in Health, Kerman University of Medical Sciences, Kerman, Iran; 4 Kerman Counseling Center for Behavioral Disorders, Kerman University of Medical Sciences, Kerman, Iran; 5 Institute for Global Health Sciences, University of California, San Francisco, California, United States of America; Infectious and Tropical Diseases Unit, Padua University Hospital, ITALY

## Abstract

**Introduction:**

HIV drug resistance (HIVDR) is an important challenge in the fight against HIV/AIDS and can threaten progress toward achieving the target of HIV elimination by 2030. Genotyping pretreatment HIVDR testing (DRT) has been proposed as a potential solution. However, the cost-effectiveness of this intervention needs to be evaluated to determine its feasibility and potential impact on healthcare systems. This study aimed to assess the cost-effectiveness of DRT among people living with HIV (PLHIV) in Iran.

**Methods:**

1000 hypothetical PLHIV were simulated in terms of cost and effectiveness based on quality-adjusted life Years (QALY). The Markov Model was developed to calculate incremental cost-effectiveness ratio (ICER) using TreeAge Pro 2020. Deterministic and probabilistic analyses were performed for sensitivity analyses.

**Results:**

Results showed that compared to not performing pretreatment HIVDR testing, this intervention gained 0.035999 QALY with an incremental cost of 1,695.32 USD. The ICER was calculated as 47,093.53 USD, indicating that pretreatment DRT was not cost-effective. The probability of opportunistic infection (OI) in people with viral failure, the effectiveness of Dolutegravir in people without drug resistance, and the quality of life (QoL) of people in the AIDS stage were found to be the most important variables affecting ICER. With an increasing willingness to pay more than 53,000 USD, pretreatment DRT testing will become cost-effective.

**Conclusion:**

Based on our findings, pretreatment HIVDR testing is not currently cost-effective in Iran as it imposes high costs on healthcare systems with few benefits for People living with HIV (PLHIV). However, if resources are available, drug resistance testing can be a valuable tool in generating HIV molecular data and molecular surveillance of HIV.

## Introduction

In 2014, the Joint United Nations Program on HIV/AIDS (UNAIDS) launched 90-90-90 targets to fight against the HIV/AIDS epidemic by 2020, and then these targets were updated to 95-95-95 by 2030. This means that by 2030, 95% of all people living with HIV (PLHIV) will know their status, 95% of all people diagnosed with HIV will receive sustained antiretroviral therapy, and 95% of all people receiving antiretroviral therapy will have viral suppression. Achieving these targets would mean that the world is on track to end the AIDS epidemic as a public health threat by 2030 [1]. In recent years, significant global progress has been made toward achieving UNAIDS targets and controlling the HIV pandemic. As of the end of 2020, 84% of PLHIV were aware of their status, and 87% of those who knew their status were receiving treatment. Among those receiving treatment, 90% had achieved viral suppression [1]. This progress toward UNAIDS targets has resulted in a significant decrease in new HIV infections and HIV-related deaths. Between 2000 and the end of 2021, new HIV infections decreased by 49%, while HIV-related deaths decreased by 61% [2].

The availability of antiretroviral therapy (ART) is one of the important factors that have resulted in a reduction in new HIV infections and HIV-related deaths. Currently, there are more than 30 antiretroviral drugs available. These drugs belong to different drug groups, including nucleoside reverse transcriptase inhibitors (NRTIs), non-nucleoside reverse transcriptase inhibitors (NNRTIs), protease inhibitors (PIs), integrase strand transfer inhibitors (INSTIs), fusion inhibitors (FI), pharmacokinetic enhancers (PE), and CCR5 antagonists [3]. In 2021, out of the 38.4 million PLHIV, 28.7 million were receiving ART (74.7% of PLHIV) (2). However, with the increasing use of ART, the prevalence of HIV drug resistance (HIVDR) has also increased. Among the 30 surveys reported from different countries to WHO, 21 showed that the prevalence of pretreatment drug resistance was more than 10% [4]. HIVDR can occur in individuals receiving ART (acquired HIVDR) or when susceptible individuals are infected with drug-resistant viruses (transmitted HIVDR) [5].

HIVDR has the potential to endanger the goal of achieving HIV elimination by 2030. Antiretroviral resistance reduces the efficacy of drugs, thereby increasing the likelihood of death and transmission of HIV to uninfected individuals. In other words, HIVDR leads to higher costs associated with HIV treatment [6]. Pretreatment HIVDR testing (DRT), which can detect transmitted HIVDR, has both individual and social benefits. At the individual level, pretreatment HIVDR testing helps physicians select the most appropriate medication regimen for the person, which can increase their lifespan and improve their quality of life (QoL). At the social level, pretreatment HIVDR testing can help prevent the transmission of the virus to uninfected people, as well as assist in selecting the best first-line medication regimen for the community. Additionally, it can be used for HIVDR surveillance purposes [7].

In Iran, approximately 49,000 people were living with HIV in 2023. Of them, 51% of them were diagnosed with HIV, 37% received antiretroviral therapy, and 33% were virally suppressed [8]. Among people who were diagnosed with HIV in Iran, the prevalence of transmitted HIV drug resistance against NNRTI drugs was 13% [9]. Since 2020, based on WHO recommendations, the first-line drugs in Iran have changed from NNRTI-based regimens to INSTIs (Dolutegravir-based regimens) [10]. Despite cohort studies reporting a low prevalence of transmitted resistance to INSTIs [11–13], increasing the use of these classes of drugs and the global prevalence of NNRTI resistance emphasizes the need to fast-track the transition to Dolutegravir-based treatments [4]. Although pre-treatment drug resistance testing is not recommended in countries where the first-line drug is Dolutegravir-based regimens, drug resistance testing can inform policymakers to change first-line drugs in a timely [6]. So, this study aimed to evaluate the cost-effectiveness of pretreatment HIV drug resistance testing in Iran.

## Materials and methods

This cost-effectiveness analysis was conducted using costs extracted from a healthcare system perspective, with a discount rate of 3% and quality-adjusted life years (QALY) measured over a 40-year time horizon. The research Ethics Committees of the Kerman University of Medical Sciences (IR.KMU.REC.1399.618) approved this study.

### Model structure

**Fig 1:**
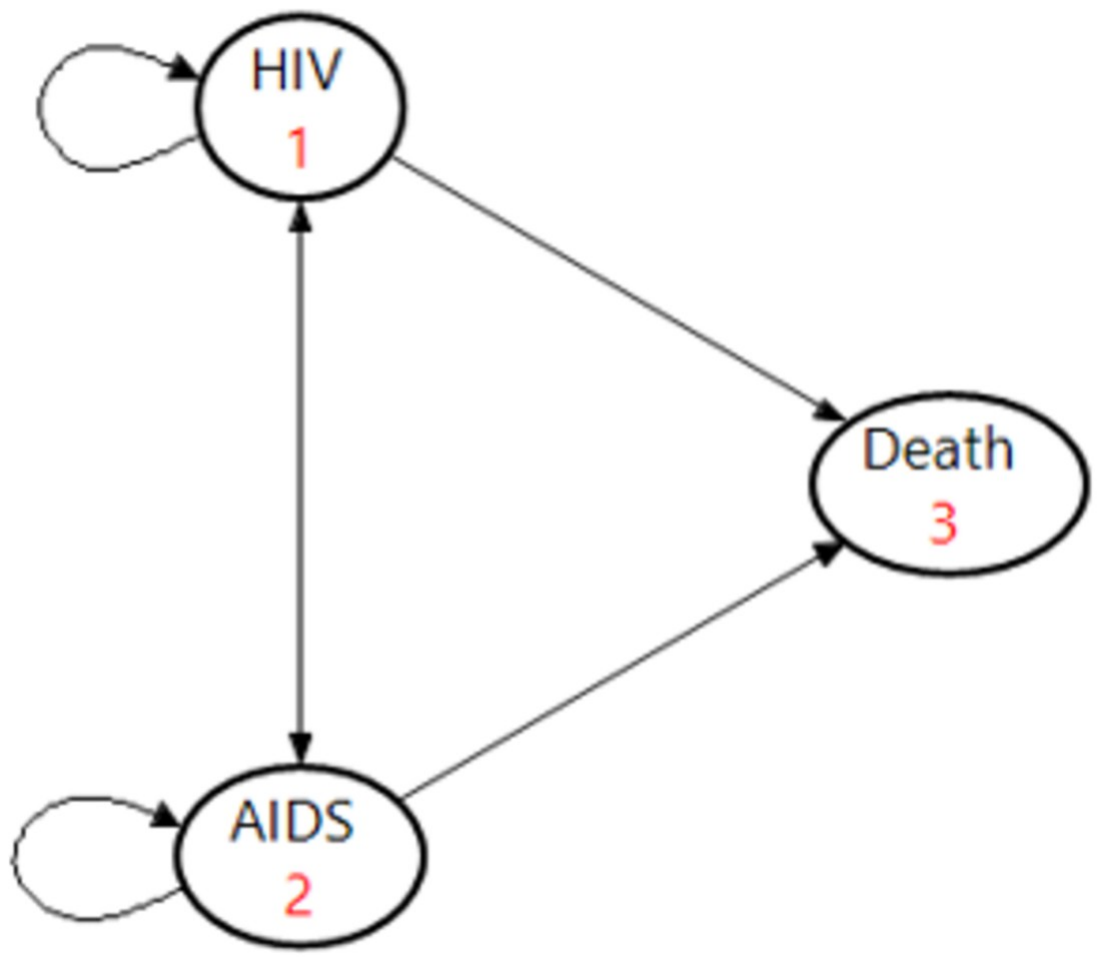
Three health states of the model.

Using a Markov model, three health states were considered for PLHIV: HIV infection, AIDS, and death. The model simulated transmission among these three states yearly for 40 years (Fig 1). 1000 hypothetical PLHIV were simulated in terms of possible care based on the condition and the stage of the disease using the PLHIV care and treatment guidelines in Iran [14].

### Strategies

**Fig 2:**
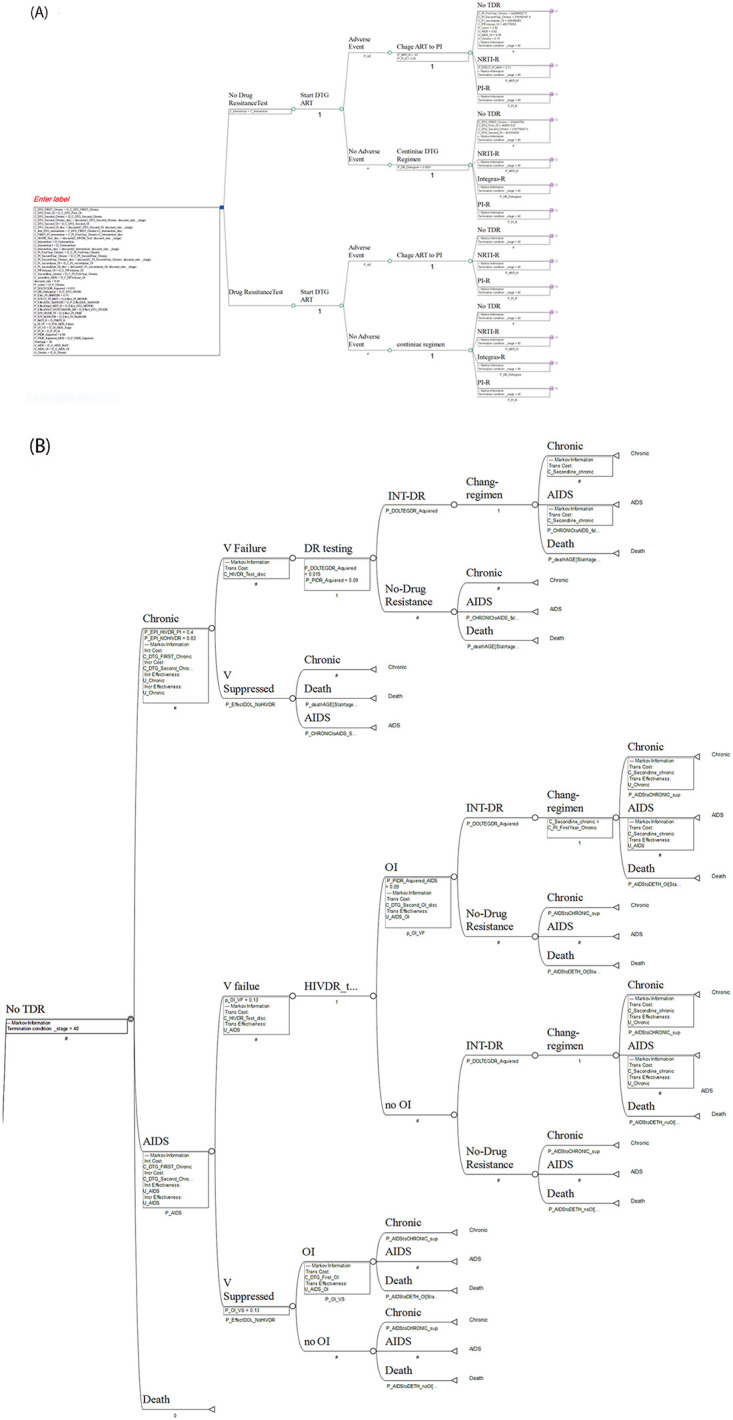
A: Markov model structure of cost-effectiveness analysis of pretreatment HIV drug resistance testing. B: Continues of the Markov model structure of cost-effectiveness analysis of pretreatment HIV drug resistance testing*. *Start DTG ART: starting treatment of PLHIV with Dolutegravir-based drugs, Change ART to PI: changing treatment regimens to protease inhibitor drugs, No TDR: People living with HIV who do not have drug resistance to treatment, NRTI-R: probability of having drug resistance to NRTI-based drugs, PI-R: probability of having drug resistance to protease inhibitor drugs, INT-DR: the probability of having drug resistance to integrase drugs, OI: the probability of having an opportunistic infection, V failure: the probability of having viral failure, V suppression: the probability of having viral suppression.

In this study, two strategies were compared, including the first strategy, doing pretreatment HIV drug resistance testing among all of the people diagnosed with HIV, and the second strategy, doing nothing to do pretreatment HIV drug resistance testing. In each strategy, treatment starts with Dolutegravir-based drugs; in drug resistance strategies, physicians will start treatment with knowledge about the presence of drug resistance, and they will prescribe drugs based on DRT results. However, in the second strategy, physicians start treatment without any knowledge about drug resistance. In this situation, the probability of treatment failure and the getting of opportunistic infection (OI) and death will be increased (Fig 2).

In each strategy, if PLHIV experienced side effects of Dolutegravir-based drugs (with a probability of 5%) [15], they should change the regimen to a protease-based regimen. In the pretreatment test strategy, the physician changes the regimen based on prior information about drug resistance; however, without test strategies, there is no prior information about drug resistance, which may result in treatment with an ineffective regimen.

In both strategies, in case of treatment failure, the drug resistance test will be taken, and they will examine for the presence of drug resistance to choose the right drugs. For people who have experienced drug resistance and treatment failure, their disease progresses, and the possibility of OIs and death increases. Therefore, the treatment regime of these people should be quickly changed to an effective drug; this drug regime in Iran is based on protease inhibitors [14].

The prevalence of resistance to NNRTI-based drugs is high (13%) [9]. Also, even in the absence of drug resistance, the probability of resistance and treatment failure for PLHIV who are under NNRTI drugs will be high. In Iran’s HIV care and treatment program, this drug group has been introduced as the last option in the treatment of PLHIV. Therefore, this drug group was not included in this modeling.

### Model parameters

#### Costs

A bottom-up approach was used to calculate the costs. In this regard, we determined the services provided to each person for one year and calculated the costs. To determine the services provided to the PLHIV, the latest national guidelines for the care and treatment of PLHIV from 2019 were used [10, 14]. We also consulted infectious disease specialists who worked on HIV treatment. Services provided to PLHIV were categorized into four groups: laboratory costs, drug costs, visits and care costs, and support costs. The frequency of some of the services (e.g., laboratory services and visits) was different in the first year after diagnosis and the later years, so we calculated costs separately for the first year and later years (Table 1).

**Table 1:** The model parameters of cost-effectiveness analysis of pretreatment HIV drug resistance testing.

	Value	Range	Reference
**Cost (**USD) **(including costs of tests, drugs, care services, and other related expenses)**			
Cost of DTG-based drugs for people in the HIV stage at first year	5,486.33		Calculated
Cost of DTG based drugs for people in the HIV stage in second year	5,018.49		Calculated
Cost of DTG-based drugs for people in the AIDS stage at first year	10,972.66		Calculated
Cost of DTG-based drugs for people in the AIDS stage at second year	10,036.98		Calculated
Cost of PI-based drugs for people in the HIV stage at first year	5,783.08		Calculated
Cost of PI-based drugs for people in the HIV stage in second year	5,195.051		Calculated
Cost of PI-based drugs for people in the AIDS stage at first year	11,566.17		Calculated
Cost of PI-based drugs for people in the AIDS stage at second year	10,390.10		Calculated
Cost of drug resistance testing using Sanger	1,181.14		Calculated
**PLHIV characteristics and probabilities**			
Mean age	36	27–45	Mirzaei H [16]
Diagnosis in HIV stage	82.3		Akbari M [17]
Diagnosis in AIDS stage	17.7		Akbari M [17]
Adverse effect of DTG	5%		Elzi L [15]
**Prevalence of drug resistance**			
Acquired HIVDR to NRTI	34%	19.0–50.0	Mirzaei H [18]
Acquired HIVDR to PI	9.0%	3.0–18.0	Mirzaei H [18]
Acquired HIVDR to INSTI	0.01	0–0.04	Mirzaei H [18]
Transmitted HIVDR to NRTI	3.0	1.0–6.0	Mirzaei H [18]
Transmitted HIVDR to PI	0	0–0.01	Mirzaei H [18]
Transmitted HIVDR to INSTI	0	0–0.08	Mirzaei H [18]
**Effectiveness of Drugs**			
Effectiveness of DTG-based drugs if there is no drug resistance	0.93	0.90–0.95	Sax PE [19]
Effectiveness of DTG-based drugs if there is resistance to NRTI	0.82	0.7–0.9	Molina J.M [20]
Effectiveness of DTG-based drugs if there is resistance to the PI	0.93	0.90–0.95	Sax PE [19]
Effectiveness of DTG-based drugs if there is resistance to INSTIs	0.35		Yiannis K [21]
Effectiveness of PI-based drugs if there is no drug resistance	0.83	0.78–0.85	Madruga JV [22]
Effectiveness of PI-based drugs if there is resistance to NRTI	0.72	0.63–79	JR Arribas [23]
Effectiveness of PI-based drugs if there is resistance to the PI	0.35		Yiannis K [21]
Effectiveness of PI-based drugs if there is resistance to INSTIs	0.83	0.78–0.85	Madruga JV [22]
Effectiveness of DTG-based drugs under DR testing	0.93	0.90–0.95	Sax PE [19]
Effectiveness of PI-based drugs under DR testing	0.83	0.78–0.85	Madruga JV [22]

All costs were converted from Iran’s rial to the US dollar at the official currency rate of 42,000 rials per dollar.

The detailed cost calculations are included in S1 File.

#### Outcome

We used QALY to measure the outcome. For this, we weighed people’s life years with QALY. To determine the QoL of PLHIV in different stages of HIV/AIDS infection, the study of Xuan et al. was used [24]. In this study, they used the visual analog scale (VAS) tool and also the EQ-5D-5L tool to evaluate the QoL of people in different stages of the disease. Based on this, the QoL for people in the HIV stage, AIDS stage without OI, and AIDS stage with OI were considered as 0.76, 0.67, and 0.58, respectively. Also, the QoL for the stage of death was considered zero.

#### Characteristics and probabilities

In this study, 1000 hypothetical PLHIV were included in the Markov model. The characteristics of the PLHIV and health outcomes were determined based on the literature review. Based on the literature review, the mean age of the people at the time of diagnosis was 36 years old, 17.7% of them were diagnosed with the AIDS stage, and 82.3% were diagnosed with the HIV stage (Table 1).

We conducted a systematic review and meta-analysis study to determine the prevalence of resistance against different antiretroviral drugs [18]. The results of the meta-analysis showed that the pooled prevalence of acquired HIVDR in people receiving ART was 34.0% (95% CI: 19.0%-50.0%) for nucleoside/nucleotide reverse transcriptase inhibitors (NRTIs), 9.0% (95% CI: 3.0% -18.0%) for protease inhibitors (PIs), 0.01% (95% CI: 0–0.04.0%) for INSTIs. The pooled prevalence of transmitted HIVDR in treatment naïve people was 3.0% (95% CI; 1.0%-6.0%) for NRTI, 5.0% (95% CI: 2.0%-9.0%) for NNRTI, and 0 for PIs and INIs. For drugs, the prevalence of drug resistance was 0. We included 0.1% (Table 1).

#### Effectiveness of the antiretroviral drugs

For PLHIV without drug resistance, the effectiveness of Dolutegravir-based drugs and protease inhibitor drugs were 93% and 83%, respectively. For PLHIV with resistance to NRTI drugs, the effectiveness of Dolutegravir-based drugs and protease inhibitor drugs was 83% and 71%, respectively. The effectiveness of Dolutegravir-based drugs and protease inhibitor drugs among PLHIV with resistance to these drugs was 35% [20–23] (Table 1).

#### Transition probabilities

The transition probability varies depending on age, stage of disease, drug resistance, and treatment failure. Transition probabilities were determined using a literature review.

In people who are in the HIV stage, the one-year probability of transition to the AIDS stage is 0.05 if there is no drug resistance and the virus is suppressed [25]. The annual probability of death in this group will be different according to the age of the people [26] (Table 2). In people with treatment failure at this stage, the probability of transition to the AIDS stage increases 1.56 times, but the probability of death does not change [27].

**Table 2:** Transition probability for people living with HIV in different stages.

Stage	Age group	Remaining in HIV stage	Transition from HIV to AIDS stage	Probability of death
People in HIV stage with suppressive virus	35–44 years old	0.946	0.05	0.004
	45–54 years old	0.933	0.05	0.017
	55–64 years old	0.937	0.05	0.013
	65 years and older	0.93	0.05	0.02
People in HIV stage with treatment failure	35–44 years old	0.944	0.05	0.006
	45–54 years old	0.924	0.05	0.026
	55–64 years old	0.93	0.05	0.02
	65 years and older	0.92	0.05	0.03
People in HIV stage without OI	35–44 years old	0.86	0.07	0.07
	45–54 years old	0.85	0.07	0.08
	55–64 years old	0.82	0.07	0.11
	65 years and older	0.77	0.07	0.16
People in HIV stage with OI	35–44 years old	0.79	0.07	0.14
	45–54 years old	0.77	0.07	0.16
	55–64 years old	0.71	0.07	0.22
	65 years and older	0.61	0.07	0.32

For people who are in the AIDS stage and do not have OIs, the probability of death in the age groups of 35 to 44 years, 45 to 54 years, 55 to 64 years, and 65 years and above was considered to be 0.07, 0.08, 0.11, and 0.16, respectively [26]. The probability of transitioning to the HIV stage in this group was considered 0.07 [28] (Table 2). For people who are in the AIDS stage, getting OI increases the probability of death by 2.1 times [29].

The probability of OI in those who fail treatment is 53%, and for those with successful treatment, it is 13% [29].

### Cost-effectiveness analysis

1000 hypothetical PLHIV were simulated in terms of possible care based on the condition and the stage of the disease using the PLHIV care and treatment guidelines in Iran. Three times the Gross Domestic Product (GDP) per capita for Iran was used for willingness-to-pay (WTP). The GDP per capita in 2020 was 2,746.4 USD for Iran, based on the information provided by the World Bank [21]. The incremental cost-effectiveness ratio (ICER) is calculated by dividing the cost difference between the two strategies (incremental cost) by the effectiveness difference between the two strategies (incremental QALY) (formula 1). The intervention is cost-effective if the ICER is less than three times GBD per capita [30]. The analysis was performed using TreeAge 2020 software.

Formula 1: Calculation of ICER

$$ICER=\frac{\Delta Cost}{\Delta Efect}=\frac{Cost\;A-Cost\;B}{Effect\;A-Effect\;B}$$

### Sensitivity analysis

We used one-way sensitivity analysis to evaluate the effect of uncertainty in input parameters, including cost, utility, and transition probabilities. We used a Beta distribution for the outcome, utility values, and transition probabilities and a Gamma distribution for costs. For sensitivity analysis, we used a Monte Carlo simulation with 10,000 times reputation.

### Ethics statement

This study was approved by the Research Ethics Committees of the Kerman University of Medical Sciences (**IR.KMU.REC.1399.618).**

## Results

The results showed that pretreatment HIV drug resistance testing was not cost-effective compared to not doing it. The ICER was 47,093.53 USD, which is more than the willingness to pay (8239.2 USD) (Table 3).

**Table 3:** The ICER of cost-effectiveness analysis of pretreatment HIV drug resistance testing.

Strategies	QALY	Incremental QALY	Cost (USD)	Incremental cost	ICER
Pretreatment HIV drug resistance testing	18.5387	0.035999	93,606.57	1,659.32	47,093.53
Doing not	18.5027		91,911.25		

### Sensitivity analysis

**Fig 3:**
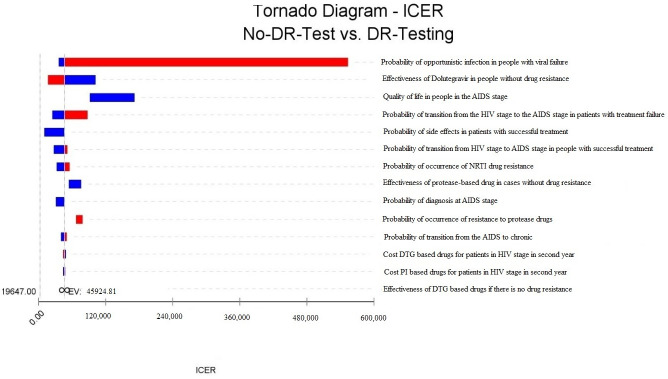
Tornado diagram of cost-effectiveness of pretreatment HIV DRT in Iran.

Deterministic sensitivity analysis using a Tornado diagram showed that the probability of OI in people with viral failure, the effectiveness of Dolutegravir in people without drug resistance, the QoL in people in the AIDS stage, the probability of transition from the HIV stage to the AIDS stage in people with treatment failure, the probability of side effects in people with successful treatment, probability of transition from HIV stage to AIDS stage in people with successful treatment, probability of occurrence of NRTI drug resistance, effectiveness of protease-based drug in cases without drug resistance, probability of diagnosis at AIDS stage, and probability of occurrence of drug resistance to protease drugs are the most important variables affecting ICER (Fig 3).

**Fig 4:**
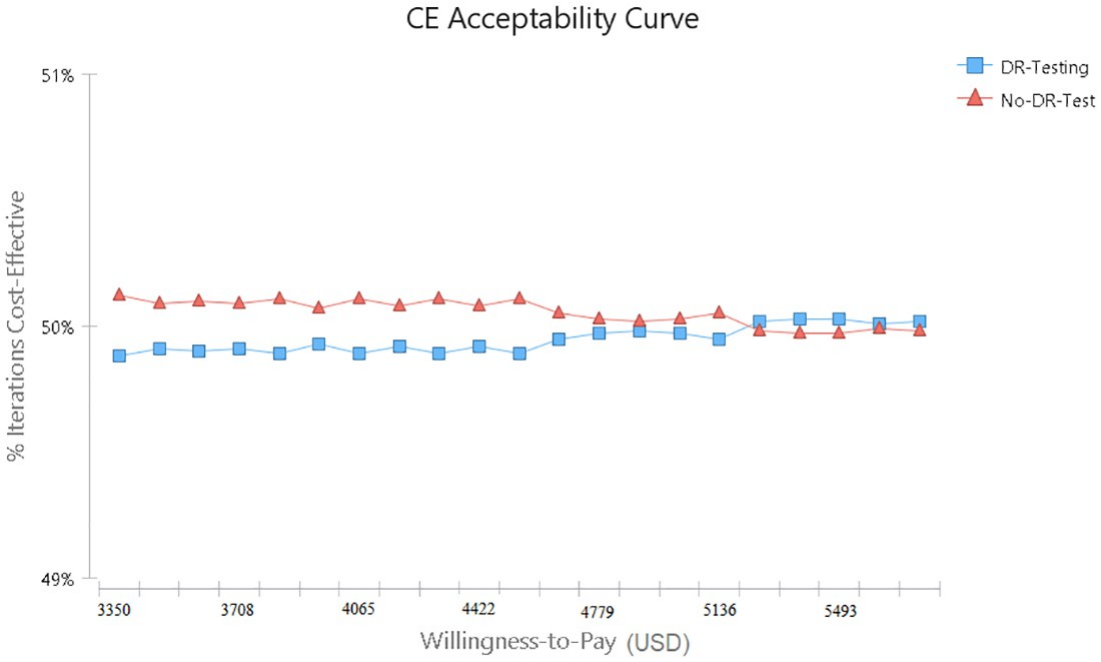
Cost-effectiveness acceptability curve.

The acceptability cure showed that the DR testing would be cost-effective with an increasing willingness to pay more than 53,000 USD (Fig 4).

**Fig 5:**
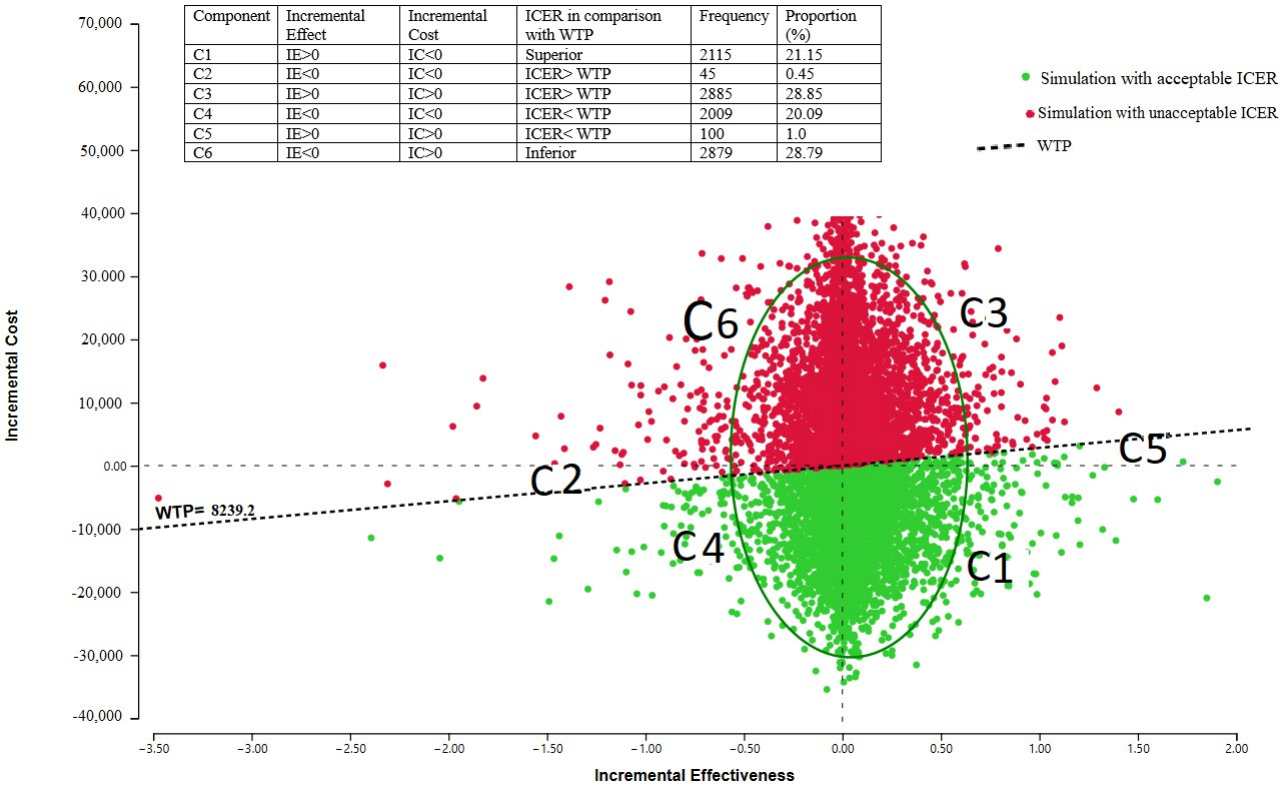
Probabilistic sensitivity analysis curve using Monte Carlo simulation.

Incremental cost-effectiveness scatter using a 1000-time Monte Carlo simulation showed that nearly 50% of the time points were in the acceptance area (Fig 5).

## Discussion

Results showed that the pretreatment HIVDR testing is not cost-effective in Iran. However, the probability of cost-effectiveness increases with increasing the willingness to pay. The most important variables that affect ICER were the probability of OI in people with viral failure, the effectiveness of Dolutegravir in people without drug resistance, the QoL in people in the AIDS stage, the probability of transition from the HIV stage to the AIDS stage in people with treatment failure, and the probability of side effects in people with successful treatment.

Results showed that the pretreatment HIVDR testing in the treatment naïve PLHIV is not cost-effective. This can be due to the low prevalence of transmitted drug resistance against DTG-based drugs in newly diagnosed people in Iran. Based on our meta-analysis study, the prevalence of pretreatment HIVDR of NRTI drugs was 3%, and for PIs and INSTIs, there was 0 [18]. In this model, there is no difference in the outcome and cost of care in the absence of drug resistance. In more than 97% of cases, there is no difference in the costs and outcomes of the people included in the model. The studies in the United States are in line with our explanation, which showed that when the first-line drugs for the treatment of HIV were NNRTI-based drugs, the pretreatment HIVDR testing was cost-effective [31]; however, when the first-line drugs changed to DTG-based drugs neither baseline HIVDR testing for NNRTI based drugs [10] nor DTG based drugs [21] were not cost-effective. Another study in sub-Saharan Africa showed that in situations where the NNRTI drugs are preferred first-line drugs, the transition to using a Dolutegravir as a first-line regimen in all new ART initiators is more cost-effective than using pretreatment HIV drug resistance testing [32]. Another explanation is that the DRT is a routine test among people who experience treatment failure in either of the two strategies. Therefore, in people who have drug resistance, even without pretreatment DRT before the start of the treatment, still drug resistance will be detected in them shortly (maximum up to one year) after the start of the treatment. This can decrease the differences in effectiveness between the two strategies.

We found that increasing the willingness to pay will make DRT cost-effective. This finding indicates the importance of the economic condition of the countries in the cost-effectiveness of the new interventions. The findings of other studies support this idea; a cost-effectiveness study in Kenya showed that even when the first-line drug was NNRTI, resistance to it was high. However, DRT in these countries was not cost-effective [33]; however, in the United States, when the first-line drug was NNRTI, DRT was cost-effective [31].

The most important variable affecting cost-effectiveness was the probability of OI in people with viral failure. High rates of OIs among people experiencing viral failure can lead to increased healthcare costs due to additional treatments, hospitalizations, and complications [34]. Pretreatment DRT can reduce the incidence of viral failure by ensuring more effective initial ART regimens, thereby lowering the overall incidence of OIs, reducing associated costs, and improving QALYs. Another factor affecting cost-effectiveness was the effectiveness of Dolutegravir in people without drug resistance. Since Dolutegravir is a potent antiretroviral with a high barrier to resistance, administering Dolutegravir can lead to better viral suppression and fewer treatment failures. This enhances outcomes and can offset the initial costs of DRT by reducing the need for subsequent, more expensive treatments and hospitalizations [11].

The QoL in people in the AIDS stage also impacts cost-effectiveness. The QoL in the AIDS stage is generally lower [24]. Pretreatment DRT can significantly improve their QoL and increase QALYs by preventing them from progressing to the AIDS stage. Another factor affecting cost-effectiveness was the probability of side effects in people with successful treatment. Even in people with successful treatment, side effects can diminish QoL and require additional medical care. People experiencing side effects from Dolutegravir-based drugs may need to change their regimen. In the pretreatment test strategy, physicians can adjust the regimen based on prior information about drug resistance. Such testing is necessary for prior information about drug resistance, which may result in treatment with an ineffective regimen. In the study of Emily in the United States [10], the most important factors were the prevalence of transmitted NRTI-R and reduced suppression of transmitted NRTI-R with a DTG-based regimen. The difference between the results of these studies can be due to different modeling methods or differences in the included parameters.

This study had four limitations. This study did not take into account the possibility of people with drug-resistant HIV transmitting the infection to uninfected individuals. Such transmission can be prevented through drug-resistance testing, which can increase the cost-effectiveness of HIVDR testing before treatment. Second, in the method of calculating the parameters of the model, many parameters have been used from other studies that were calculated in different conditions, and they may not match the actual value of the parameter in Iran. To cover this limitation, we tried to do a Sensitivity analysis to solve this problem. Third, we considered only the clinical benefits of DRT for individual PLHIV. At the same time, DRT has public health benefits, e.g., the molecular data extracted for DRT can be used for molecular surveillance of HIV, and this molecular data can be used to detect HIV transmission clusters. Fourth, we attempted to minimize the number of parameters to simplify the model. For instance, in the event of virological failure on a dolutegravir-based regimen, we switched the treatment to just a PI regimen. However, initiating an optimized regimen that does not include PIs in all cases is possible. This potential strategy could impact overall costs and should be considered in future analyses.

## Conclusion

The study findings indicate that, given the present circumstances in Iran, conducting pretreatment HIVDR testing is not deemed cost-effective. This intervention places a substantial financial burden on the healthcare system while yielding limited benefits for PLHIV. Therefore, its implementation is not advised under conditions of constrained resources. However, in cases where resources are more abundant, drug resistance testing can prove highly valuable for generating HIV molecular data and facilitating molecular surveillance of HIV.

## Supporting information

S1 File Detailed cost calculations. (DOCX)
