# Supplementary material for: Cost-effectiveness of pretreatment HIV drug resistance testing in people living with HIV in Iran
Source: PLoS One. 2024 Sep 6;19(9):e0309528. doi: 10.1371/journal.pone.0309528 (PMC11379287; doi:10.1371/journal.pone.0309528)
Supplement: S1 File — (DOCX) [file pone.0309528.s001.docx]

Supplementary: The detailed cost calculations


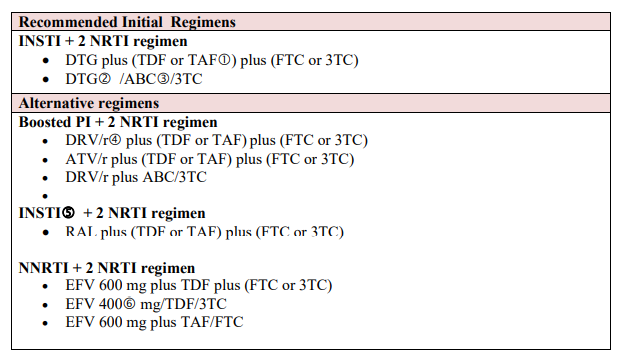


Figure 1: Recommended and alternative regimen based on Iran’s national guideline for the care and treatment of PLHIV

Source of figure: National guideline for the care and treatment of PLHIV

Table 1: Unit and annual price of recommended initial HIV drugs in Iran (USD)

| Drugs | Frequency | Unit price | Total price for one year (USD) |
| --- | --- | --- | --- |
| DOLUTEGRAVIR (DTG) | Once a day | 0.67 | 245.94 |
| TENOFOVIR (TDF) | Once a day | 1.31 | 477.98 |
| EMTRICITABINE (FTC) | Once a day | 1.19 | 434.52 |
| total |  |  | 1158.44 |

Table 2: Unit and annual price of alternative initial HIV drugs in Iran (USD)

| Drugs | Frequency | Unit price | Total price for one year (USD) |
| --- | --- | --- | --- |
| DRV/r | Once a day | 1.64 | 599.64 |
| TENOFOVIR (TDF) | Once a day | 1.31 | 477.98 |
| EMTRICITABINE (FTC) | Once a day | 1.19 | 434.52 |
| total |  |  | 1512.14 |

Table 3: Costs of tests related to people who are living with HIV (PLHIV) per patient (USD)

|  | frequency | Unit price | Total price for one year (USD) |
| --- | --- | --- | --- |
| CD4 | 6 months | 61.48 | 122.95 |
| Viral load | 6 months | 642.14 | 1,284.29 |
| Drug resistance testing | In case of treatment failure |  | - |
| Pregnancy test in women | before starting treatment | 59.52 | 24.11 |
| HBs Ag | Every 12 months, if the previous test is negative | 29.15 | 24.78 |
| HBsAb | Every 12 months, if the previous test is negative | 29.15 | 24.78 |
| HBcab | Every 12 months, if the previous test is negative | 29.15 | 24.78 |
| VDRL | Every 12 months, if the previous test is negative | 3.78 | 3.22 |
| PPD | Every 12 months, if the previous test is negative | 1.71 | 1.45 |
| Pap smeer | Once a year | 98.36 | 44.26 |
| 0ther tests |  |  | 100 |
| Total |  |  | 1,654 |

Table 4: The cost of care services to PLHIV in Iran (USD)

|  | Frequency in first year | Frequency in next years | Costs per case |
| --- | --- | --- | --- |
| Visit and consultation by general practitioner | 2 times a month in the first two months and once a month until the end of the first year | 6 times a year | 16.43 |
| Counseling by a psychologist | 2 times a month in the first two months and once a month until the end of the first year | 6 times a year | 11.43 |
| Counseling by health care providers and nurse | 2 times a month in the first two months and once a month until the end of the first year | 6 times a year | 11.43 |
| Infectious specialist visit | Once a year | One a year | 24.76 |
| Psychiatrist visit | Once a year | Once a year | 32.86 |
| Gynecologist visit | Once a year | Once a year | 24.76 |
| Dentistry | If needed | If needed | 238.1 |
| Condom | 12 packages per year | 12 packages per year | 7.15 |
| Total cost for a year | 1485.28 | 1372.42 |  |

Maintenance cost

The cost of maintenance includes the cost of building, water, electricity, gas, telephone, cleaning, and maintenance, which was considered an average of 20 million Rials (476.19 USD) per person per year. The opinions of the person in charge of the counseling center for behavioral diseases were used for maintenance costs.

In addition, according to experts, 30 million Rials (714.28 USD) were added as other expenses.
